# Gene selection with multiple ordering criteria

**DOI:** 10.1186/1471-2105-8-74

**Published:** 2007-03-05

**Authors:** James J Chen, Chen-An Tsai, ShengLi Tzeng, Chun-Houh Chen

**Affiliations:** 1 Division of Biometry and Risk Assessment, National Center for Toxicological Research, Food and Drug Administration, Jefferson, Arkansas, USA; 2 Institute of Statistical Science, Academia Sinica, 128, Academia Rd. Sec. 2, Taipei 115, Taiwan

## Abstract

**Background:**

A microarray study may select different differentially expressed gene sets because of different selection criteria. For example, the fold-change and p-value are two commonly known criteria to select differentially expressed genes under two experimental conditions. These two selection criteria often result in incompatible selected gene sets. Also, in a two-factor, say, treatment by time experiment, the investigator may be interested in one gene list that responds to both treatment and time effects.

**Results:**

We propose three layer ranking algorithms, point-admissible, line-admissible (convex), and Pareto, to provide a preference gene list from multiple gene lists generated by different ranking criteria. Using the public colon data as an example, the layer ranking algorithms are applied to the three univariate ranking criteria, fold-change, p-value, and frequency of selections by the SVM-RFE classifier. A simulation experiment shows that for experiments with small or moderate sample sizes (less than 20 per group) and detecting a 4-fold change or less, the two-dimensional (p-value and fold-change) convex layer ranking selects differentially expressed genes with generally lower FDR and higher power than the standard p-value ranking. Three applications are presented. The first application illustrates a use of the layer rankings to potentially improve predictive accuracy. The second application illustrates an application to a two-factor experiment involving two dose levels and two time points. The layer rankings are applied to selecting differentially expressed genes relating to the dose and time effects. In the third application, the layer rankings are applied to a benchmark data set consisting of three dilution concentrations to provide a ranking system from a long list of differentially expressed genes generated from the three dilution concentrations.

**Conclusion:**

The layer ranking algorithms are useful to help investigators in selecting the most promising genes from multiple gene lists generated by different filter, normalization, or analysis methods for various objectives.

## Background

Recent advances in DNA microarray technology provide exciting tools for studying the expression levels of thousands of distinct genes simultaneously. A common data analysis approach is to identify a subset of key genes from the original gene set that express differentially under different experimental conditions with a goal to determine the underlying relationship between samples and genes or gene clusters. The relationship is used to identify biological functions or to predict specific biological or therapeutic outcomes from the subset of key genes. Selection of differentially expressed genes can be separated into two steps. The first step is to calculate a discriminatory score that will rank the genes in order of evidence of differential expressions. The second step is to determine a cutoff (threshold) from the ranked scores to divide the genes into two lists: the differentially expressed and the non-differentially expressed genes. The genes above the threshold are selected as differential expressions. Criteria for determining the threshold cutoff should depend on the objective of the experiment. For instance, if the objective is to identify a small number of truly differentially expressed genes for further study, then a stringent criterion such as controlling either the familywise or the false discovery error rate may be appropriate. However, if the purpose is to determine functional relationships among genes that have been affected by treatments or to develop a genomic biomarker classifier, criteria that do not eliminate as many genes may be more appropriate since the omission of informative genes would have a much more serious consequence than the inclusion of non-informative genes. In all applications, the first step of gene ranking is the more important of the two. Fold-change and p-value are two common approaches to selecting differentially expressed genes when the experiment consists of two conditions (normal versus tumor). In the fold-change approach, a gene is said to be differentially expressed if the ratio in absolute value of the expression levels between the two classes exceeds a certain threshold, e.g., a 2-fold or 3-fold change. These genes are selected as differential expressions. This approach is deficient in some aspects as it does not account for the variability of the expression levels among genes. For example, genes with larger variances have a good chance of exhibiting larger fold-changes even if they are not differentially expressed. The p-value ranking is an alternative approach for gene ranking. The p-value is the probability outcome from a statistical testing procedure that there is no difference between two conditions for an individual gene. A small p-value is evidence of differential expressions. One common problem encountered in the use of the p-value ranking is that a gene with small fold change can have a very small p-value (below the p-value threshold) because of a very small standard deviation. These two ranking criteria often result in selecting different lists of differentially expressed genes.

One important application in microarray experiments is to develop a prediction model to discriminate different biologic phenotypes or to predict the diagnostic category or prognostic stage of a patient. Because thousands of gene are involved, many genes are often noisy in nature and many are irrelevant for prediction; the use of all predictors can suppress or reduce the performance of a classification algorithm. The prediction model is often based on a selected gene set from a p-value ranking criterion (e.g., [1]). Alternatively, genes can be ranked according to its predictive accuracy (discriminatory ability) by performing gene-by-gene prediction. The wrapper approach is an alternative gene selection method; the wrapper approach finds a subset of genes and evaluates its relevance while building the prediction model. For example, the classification tree (CTree) constructs a binary hierarchical classifier [2] through recursively partitioning parent nodes into two child nodes. In each node, CTree searches all possible predictors and selects the predictors that minimize overall true impurity. Guyon et al. [3] proposed a recursive feature elimination (RFE) procedure for the support vector machine (SVM) classification algorithm. The SVM-RFE method uses the magnitude as a ranking criterion to select feature predictors. A strategy of applying the wrapper approach to gene ranking is to examine the frequency of selections in the cross validation [4,5]. The most frequently selected genes are presumed to be most relevant to the sample distinction. The gene ranking criteria described above for prediction purposes would result in different lists; also, these ranking criteria are different from either the fold-change or p-value ranking criterion. As discussed, there are two general objectives in gene selection. One objective is to develop a classifier or predictive model for class prediction, and the other is to identify differentially expressed genes for a follow-up study. These two objectives are not mutually exclusive. For example, the set of differentially expressed genes identified presumably for the second objective can be used to develop a classifier (e.g., [1,6]) for the first objective. Different gene selection procedures often result in different rankings, even for the same objective such as the p-value and fold-change criteria to identify differentially expressed genes. Because of thousands of genes involved and difficulty in the validation, a gene ranking procedure that strikes a balance among several ordering criteria will be useful for microarray data analysis. Furthermore, many microarray experiments have involved two or more factors and/or more than two experimental conditions. In the Applications section below, we consider a two-factor microarray study to identify biological effects of radiation exposure on gene expression. The experiment consists a control and dose groups. The RNA samples are extracted from the control and exposed cells at 4 hours and 24 hours. Statistical analysis would consist of a comparison between control and dose groups to investigate the radiation effect on gene expression, and a comparison between the two time points to investigate the time effect. Each comparison will result in one gene list according to the p-values from the respective statistical test. A gene list that accounts for both ranking criterions will be useful for investigating the most important genes that respond to the treatment effect as well as time effect.

Given the wide uses of microarray technology, selection of differential expressed genes is one of the most important objectives in microarray data analysis. In a simple experiment with two experimental conditions, different experimental objectives or different analysis methods can generate different lists of differentially expressed genes. For experiments with more than two experimental conditions, the analysis will generate different gene lists from different test hypotheses.

This paper proposes three layer ranking algorithms for gene ranking with multiple ranking criteria, where each individual criterion constitutes its ordering of preference for selection. The presentation is limited to two and three univariate ranking criteria.

## Results

### Example

The colon cancer data set [7] consists of 2000 human genes with highest minimal intensity across 40 tumor and 22 normal colon tissue samples. A goal of a data analysis is to select a set of genes that express differently between the normal samples and cancer samples. We consider three criteria to select the set of marker genes: fold-change, p-value, and frequency of selections by the SVM-RFE classifier. The fold-change and p-value were computed for each gene. The p-values were computed based on 100,000 permutations using the t-statistic with unequal variances. When a tie occurs, the t-statistic is used to break the tie. The SVM-RFE method was used to rank the discriminatory power of a gene using a 10-fold cross-validation method. Briefly, the entire data set was divided into 10 subsets (6 or 7 samples per subset) of roughly equal size. The SVM-RFE was trained with a selection of 'eight' optimal genes on the 9 (= 10-1) subsets (either 56 or 55 samples) together and then applied to the remaining subset as the test data set.

The classification rule is iterated 10 times to complete an analysis of entire data set. The entire process was repeated 250 times each time the 62 samples were randomly partitioned into 10 subsets. The frequency of selections for the 2000 genes over the 2500 replicates were calculated as the 3rd selection criterion.

The colon data set has been analyzed extensively by many researchers using various gene selection and/or classification procedures (e.g., [5,6,8,9]). The classification accuracy rates reported from various V-fold cross-validations, where V = 2, 3, 5, 10, and 62, are between 70% to 89%. The average accuracy rate in our 250 10-fold cross-validations by the SVM-RFE is 84.6% for selecting 8 genes. The accuracy rates are 85.3% and 87.6% for selecting 16 and 32 genes, respectively. These accuracy rates are comparable or better than most of reported results in the literature.

Table 1 shows the p-values (P-val), fold-change (FC), frequency (FQ) and their univariate rankings for the top 50 p-value ranked genes. Note that the frequency ranking only has 47 categories; 1926 genes have never been selected. It can be seen that the three ranking criteria give very different gene lists. Among those top 50 p-value ranked genes, 29 and 21 are in the top 50 lists ranked by the fold-change and frequency, respectively. The fold-change and p-value rankings have a better agreement because they both measure the fold-change except that p-value ranking adjusts for the variability of the fold-change across arrays.

**Table 1:** One-Dimensional Ranks for Colon Data set

P-value 1–25 ranked genes							P-value 26–50 ranked genes						
	
Raw Scores				Ranks			Raw Scores				Ranks		
			
Gene	P-v	FC	FQ	P-v	FC	FQ	Gene	P-v	FC	FQ	P-v	FC	FQ
R87126	1.0E-6	-2.89	2360	1	12	2	T57619	1.2E-4	1.70	0	26	186	47
R36977	1.0E-6	2.62	404	2	16	13	D31885	1.2E-4	2.15	0	27	28	47
M22382	2.0E-6	2.49	2	3	24	45	T86749	1.2E-4	2.27	0	28	38	47
M26383	3.0E-6	4.01	2436	4	2	1	X56597	1.2E-4	2.42	0	29	45	47
H08393	4.0E-6	2.34	2339	5	35	3	U26312	1.3E-4	2.49	0	30	25	47
X12671	5.0E-6	2.74	98	6	14	25	X55715	1.3E-4	1.86	1	31	120	46
X63629	6.0E-6	2.50	360	7	23	14	T95018	1.5E-4	1.80	0	32	139	47
M63391	9.0E-6	-3.59	1409	8	4	5	R84411	1.6E-4	2.52	62	33	20	30
Z50753	9.0E-6	-1.94	1375	9	94	6	M36981	1.7E-4	1.95	0	34	87	47
J02854	1.1E-5	-4.23	214	10	1	17	T47377	1.8E-4	3.72	290	35	3	15
													
H43887	1.3E-5	-3.26	88	11	6	27	U17899	1.9E-4	2.31	2	36	37	45
J50302	1.4E-5	2.62	117	12	17	22	U51023	2.0E-4	2.00	0	37	75	47
H40095	1.7E-5	2.41	2	13	29	45	T62947	2.1E-4	2.03	579	38	64	10
M36634	1.8E-5	-2.60	12	14	18	39	R42501	2.2E-4	1.84	0	39	126	47
T86473	1.9E-5	2.39	0	15	33	47	M76378	2.3E-4	-2.44	11	40	27	40
U09564	2.0E-5	2.12	0	16	50	47	T92451	2.6E-4	-3.21	2	41	8	45
U30825	2.3E-5	1.79	0	17	141	47	R64115	2.9E-4	1.98	0	42	79	47
X14958	2.5E-5	2.00	0	18	72	47	T51261	2.9E-4	2.09	1106	43	53	8
M26697	3.2E-5	2.10	0	19	52	47	X86693	3.0E-4	-3.28	1	44	5	46
X54942	4.6E-5	2.64	0	20	15	47	T61609	3.1E-4	1.71	0	45	183	47
													
M76378	5.3E-5	-3.24	496	21	7	12	T48804	3.4E-4	1.69	0	46	194	47
T71025	7.5E-5	-1.83	19	22	128	35	T51529	3.4E-4	1.66	0	47	211	47
T56604	8.6E-5	1.89	0	23	110	47	H55758	3.6E-4	1.76	0	48	149	47
H06524	1.0E-4	-3.09	1281	24	9	7	T58861	3.7E-4	1.81	1	49	135	46
M76383	1.0E-4	-2.52	54	25	21	31	X70326	3.8E-4	2.12	1	50	51	46

Gene ID, p-value (P-v), fold-change (FC), frequency (FQ) and their univariate ranks ordered by the p-value (t-statistic) rank for the top 50 ranked genes from the 2,000 genes in the colon data set. The p-values are computed based on 100,000 permutations.

#### Two-Dimensional Layer Ranking: Fold-change and p-value

**Figure 1:**
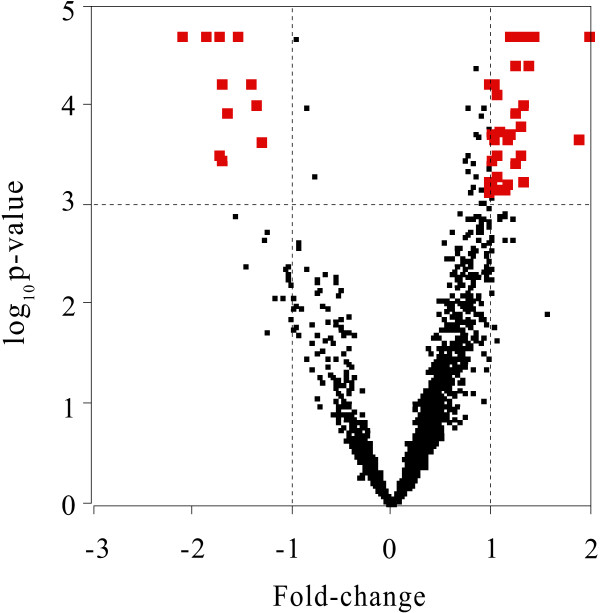
**Volcano Plot for Colon Data set**. Volcano plot for the 2000 genes from the colon cancer data. The x-axis is the fold-change value and y axis is - log_10 _p-value, based on 50,000 permutations. Using the absolute 2-fold change and p-value 0.001 as the threshold cutoff, forty five genes in the upper left and upper right are selected.

The volcano plot (e.g., [10]) has been used to summarize the two ranking schemes. The volcano plot is a plot of the p-value (-log_10_*p*) versus fold-change (FC). The genes on the upper left or upper right corners represent small p-vales with large fold changes (Figure 1). On the other hand, the genes on the upper middle have small fold-changes, but, having small p-values, these genes will not be on the top list by the fold-change ranking. Similarly, the gene on the lower left or lower right have large fold-changes, but, with larger p-values, these genes will not be on the top list by the p-value ranking. The layer-ranking algorithms take all these genes into account.

Applying layer ranking procedures, the numbers of layers obtained from the point-admissible, convex, and Pareto algorithms are 1268, 403, and 206, respectively. Table 2 lists the top 49 genes according to the point-admissible ranking (the next layer consists of 4 genes). The three layer rankings generally show good agreements except that some genes may rank one layer higher or lower. Note that the differences between two adjacent gene rankings are no more than 2. For the 49 genes, the numbers of layers for the point-admissible, convex, and Pareto algorithms are 26, 13, and 10, respectively. The 49 genes include all top 29 ranked genes by the fold-change ranking, top 31 by the p-value. Table 2 shows some differences between the layer rankings and the p-value ranking or the fold-change ranking. Gene T47377, which is ranked 35th by the p-value having a large fold-change 3.72 (rank 3rd), is ranked 8th by the point-admissible (3rd layer) and Pareto (2nd layer) algorithms and is ranked 6th (2nd layer) by the convex algorithm. On the other hand, gene H08393 has a small p-value (ranked 5th) with small fold-change 2.34 (ranked 35th), and is ranked in the 12, 11, and 10 by the three layer algorithms.

**Table 2:** Comparison of Two-Dimensional Ranking for Colon Data set

Point-Admissible top 1–25 layer ranked genes						Point-Admissible top 26–49 layer ranked genes					
	
Gene	P-A	Convex	Pareto	P-v	FC	Gene	P-A	Convex	Pareto	P-v	FC
R87126	1	1	1	1	12	R84411	13	7	6	33	20
M26383	1	1	1	4	2	U09564	14	8	7	16	50
J02854	1	1	1	10	1	M26697	15	9	8	19	52
R36977	1	2	1	2	16	X14958	15	9	8	18	72
M63391	2	2	2	8	4	U26312	15	8	7	30	25
M22382	3	3	2	3	24	R54097	15	8	6	54	19
X12671	3	3	2	6	14	X56597	15	9	7	29	28
T47377	3	2	2	35	3	U30825	16	9	8	17	141
H43887	4	3	3	11	6	R08183	17	9	7	51	22
M76378	5	4	4	21	7	M76378	18	10	8	40	27
											
X86693	5	3	3	44	5	D31885	18	11	7	27	45
X63629	5	4	3	7	23	T86749	18	10	7	28	38
H08393	5	4	3	5	35	T71025	19	10	9	22	128
H06524	6	5	5	24	9	T56604	19	11	9	23	110
T92451	7	5	5	41	8	U17899	20	11	8	36	37
X54942	7	6	4	20	15	T60155	21	10	8	101	26
Z50753	8	5	4	9	94	X12466	22	11	9	53	32
J05032	8	5	4	12	17	T57619	23	12	10	26	186
M36634	9	6	5	14	18	M36981	24	12	9	34	87
M27190	10	6	6	233	10	H77597	24	11	9	86	31
											
T60778	10	6	6	77	11	X55715	25	13	10	31	120
H40095	11	6	5	13	29	T62947	25	12	9	38	64
L05144	12	7	7	117	13	T51023	26	13	9	37	75
M76378	13	7	6	25	21	H11084	26	12	10	83	34
T86473	13	7	6	15	33						

Gene ID, point-admissible (P-A), Convex, and Pareto layer rankings, and the p-value (P-v) and fold-change (FC) rankings for the top 49 genes selected based on the point-admissible ranking from the 2,000 genes in the colon data set.

#### Three-Dimensional Layer Ranking: Fold-change, p-value, and frequency

The numbers of layers obtained from the 3-dimensional point-admissible, convex, and Pareto algorithms are 74, 394, and 11, respectively. The convex ranking produces more layers than the point-admissible due to the discreetness of the frequency that 1926 genes (all ranked 47th) has 0 occurrence. In general, as the dimension increases, the number of layers decrease.

Table 3 lists the top 46 genes according to the point-admissible ranking (the next layer consists of 6 genes). For the 46 genes, the numbers of layers for the point-admissible, convex, and Pareto algorithms are 15, 9, and 5, respectively. The three layer rankings generally show good agreements. The differences between two adjacent gene rankings are no more than 2. Similar to the 2-dimensional layer ranking, genes that are ranked first in the one-dimension are ranked in the first layer in the 3-dimension. The 46 genes include all top 25 ranked genes by the univariate p-value ranking, top 25 by the fold-change, and top 17 by the frequency. Furthermore, 39 of the 46 genes are selected by the fold-change and p-value criteria shown in Table 2. The other 7 genes are L11706, T51261, H55916, J03210, U22055, H64489 and R62549, they all have high p-values and low fold-changes, but, higher frequencies.

**Table 3:** Comparison of Three-Dimensional Ranking for Colon Data set

Point-Admissible top 1–25 layer ranked genes							Point-Admissible top 26–46 ranked genes						
	
Gene	P-A	Convex	Pareto	P-v	FC	FQ	Gene	P-A	Convex	Pareto	P-v	FC	FQ
R87126	1	1	1	1	12	2	H40095	9	5	4	13	29	45
M26383	1	1	1	4	2	1	H55916	9	6	4	78	47	16
J02854	1	1	1	10	1	17	L05144	10	6	5	117	13	47
R36977	1	2	1	2	16	13	M76378	10	6	4	25	21	31
M63391	2	2	2	8	4	5	R84411	10	6	4	33	20	30
T47377	2	2	2	35	3	15	J03210	10	6	5	610	584	11
M22382	3	3	2	3	24	45	R54097	11	7	4	54	19	33
X12671	3	3	2	6	14	25	T86473	12	6	5	15	33	47
M76378	3	3	3	21	7	12	T71025	12	7	4	22	128	35
H08393	3	2	2	5	35	3	U22055	12	6	5	66	69	20
													
H06524	3	3	3	24	9	7	U09564	13	7	5	16	50	47
H43887	4	3	3	11	6	27	M26697	13	8	5	19	52	47
X63629	4	4	2	7	23	14	X14958	13	8	5	18	72	47
Z50753	4	3	3	9	94	6	U26312	14	7	5	30	25	47
L11706	4	3	3	135	111	4	X56597	14	8	5	29	28	47
X86693	5	3	3	44	5	46	U30825	14	8	5	17	141	47
H11084	5	5	4	83	34	9	H64489	14	8	5	121	57	24
J05032	6	4	3	12	17	22	R62549	14	7	5	82	124	19
T62947	6	5	4	38	64	10	R08183	15	8	5	51	22	46
T51261	6	4	4	43	53	8	M76378	15	8	5	40	27	40
													
T92451	7	4	4	41	8	45	T56604	15	9	5	23	110	47
X54942	7	5	4	20	15	47							
M36634	8	5	4	14	18	39							
M27190	9	5	5	233	10	47							
T60778	9	5	5	77	11	47							

Gene ID, point-admissible (P-A), Convex, and Pareto layer rankings, and the p-value (P-v), fold-change (FC), frequency (FQ) rankings for the top 46 genes selected based on the point-admissible ranking from the 2,000 genes in the colon data set.

Both gene T47377 (a larger p-value) and gene H08393 (a smaller fold-change) discussed previously in Table 2 have high frequencies (Table 1), both are ranked in the top 10 in Table 3. Gene D31885 and gene T86749, which are ranked in layer #18 in Table 2, are not listed in Table 3 because of 0 frequency.

### Simulation Experiment

We conducted a simulation experiment to compare the two-dimensional (p-value and fold-change) layer rankings to the univariate p-value ranking for selection of differentially expressed genes. The top-ranked genes from the univariate p-value ranking and the three layer rankings were evaluated based on the false discovery rate (FDR) of 5% [11]. The experiment considered to *m *= 1000 genes, in which *m*_1 _= 50 or 100 genes were differentially expressed with a constant effect size (*δ*), ranging from 1.0 to 2.0 with an increment of 0.2, as well as 2.5 and 3.0. Note that the *δ *of 1 represents a 2-fold change. The number of arrays in each group was 10 or 15. The data were sampled from a normal distribution under an independent model or a correlated model. For the correlated model, we considered a block compound symmetry (CS) correlation structure [12], in which there were 100 blocks and each block consists of 10 dependent genes with a pairwise correlation coefficient *ρ*. We assumed that the first *m*_1_/10 blocks corresponded to the *m*_1 _differentially expressed genes. Therefore, the *m *× *m *variance-covariance matrix **Σ **of a block CS structure for the simulation model consisted of 100 equal blocks, and each block Σ_*i *_has a CS structure with variances of 1 and a common correlation *ρ*; that is,

Σ=[Σ10⋯00Σ2⋯0⋮⋮⋱⋮00⋯Σ100],
 MathType@MTEF@5@5@+=feaafiart1ev1aaatCvAUfKttLearuWrP9MDH5MBPbIqV92AaeXatLxBI9gBaebbnrfifHhDYfgasaacH8akY=wiFfYdH8Gipec8Eeeu0xXdbba9frFj0=OqFfea0dXdd9vqai=hGuQ8kuc9pgc9s8qqaq=dirpe0xb9q8qiLsFr0=vr0=vr0dc8meaabaqaciaacaGaaeqabaqabeGadaaakeaaiiqacqWFJoWucqGH9aqpdaWadaqaauaabeqaeqaaaaaabaGaeu4Odm1aaSbaaSqaaiabigdaXaqabaaakeaacqaIWaamaeaacqWIVlctaeaacqaIWaamaeaacqaIWaamaeaacqqHJoWudaWgaaWcbaGaeGOmaidabeaaaOqaaiabl+UimbqaaiabicdaWaqaaiabl6Uinbqaaiabl6UinbqaaiablgVipbqaaiabl6UinbqaaiabicdaWaqaaiabicdaWaqaaiabl+Uimbqaaiabfo6atnaaBaaaleaacqaIXaqmcqaIWaamcqaIWaamaeqaaaaaaOGaay5waiaaw2faaiabcYcaSaaa@4F24@

where each Σ_*i *_had an equicorrelated structure,

Σi=[1ρ⋯ρρ1⋯ρ⋮⋮⋱⋮ρρ⋯1],
 MathType@MTEF@5@5@+=feaafiart1ev1aaatCvAUfKttLearuWrP9MDH5MBPbIqV92AaeXatLxBI9gBaebbnrfifHhDYfgasaacH8akY=wiFfYdH8Gipec8Eeeu0xXdbba9frFj0=OqFfea0dXdd9vqai=hGuQ8kuc9pgc9s8qqaq=dirpe0xb9q8qiLsFr0=vr0=vr0dc8meaabaqaciaacaGaaeqabaqabeGadaaakeaacqqHJoWudaWgaaWcbaGaemyAaKgabeaakiabg2da9maadmaabaqbaeqabqabaaaaaeaacqaIXaqmaeaaiiGacqWFbpGCaeaacqWIVlctaeaacqWFbpGCaeaacqWFbpGCaeaacqaIXaqmaeaacqWIVlctaeaacqWFbpGCaeaacqWIUlstaeaacqWIUlstaeaacqWIXlYtaeaacqWIUlstaeaacqWFbpGCaeaacqWFbpGCaeaacqWIVlctaeaacqaIXaqmaaaacaGLBbGaayzxaaGaeiilaWcaaa@4E7E@

where *ρ *= 0.3 or 0.6 was the common correlation coefficient. In each array, expression data for the non-differentially expressed genes in each block were generated from the multivariate normal distribution *N*(**0**, Σ_*i*_), and expression data for the differentially expressed genes in each block were generated from the multivariate normal distribution *N*(*δ***1**, Σ_*i*_), where **1 **denoted a 10-dimensional unity vector. For each simulated data set, the p-values and fold changes were computed. Three layer-ranked gene lists were then generated. The p-values were computed based on t-statistic from 50,000 random permutations. The simulation was repeated 500 times for each combination of *m*_1_, *n*, *δ*, and *ρ*.

We evaluated the same number of top ranked genes selected by the p-value ranking and by the three layer rankings in terms of the control of the FDR and the ability (power) to detect differential expression. The four ranking procedures were evaluated as follows. First, the differentially expressed genes from the p-value ranking were selected using the Benjamini and Hochberg (BH) FDR-controlled procedure [11]. The same number of top ranked genes, then, was selected as differentially expressed genes for each layer ranking. For each selected gene set in each simulation, the numbers of false positives (*V*_*k*_) and true positives (*T*_*k*_) were counted, *k *= 1, ..., 500. The FDR and the power were estimated by

FDR=1500∑k=1500VkVk+TkandPower=1500×m1∑k=1500Tk.
 MathType@MTEF@5@5@+=feaafiart1ev1aaatCvAUfKttLearuWrP9MDH5MBPbIqV92AaeXatLxBI9gBaebbnrfifHhDYfgasaacH8akY=wiFfYdH8Gipec8Eeeu0xXdbba9frFj0=OqFfea0dXdd9vqai=hGuQ8kuc9pgc9s8qqaq=dirpe0xb9q8qiLsFr0=vr0=vr0dc8meaabaqaciaacaGaaeqabaqabeGadaaakeaafaqabeqadaaabaGaeeOrayKaeeiraqKaeeOuaiLaeyypa0ZaaSaaaeaacqaIXaqmaeaacqaI1aqncqaIWaamcqaIWaamaaWaaabCaeaadaWcaaqaaiabdAfawnaaBaaaleaacqWGRbWAaeqaaaGcbaGaemOvay1aaSbaaSqaaiabdUgaRbqabaGccqGHRaWkcqWGubavdaWgaaWcbaGaem4AaSgabeaaaaaabaGaem4AaSMaeyypa0JaeGymaedabaGaeGynauJaeGimaaJaeGimaadaniabggHiLdaakeaacqqGHbqycqqGUbGBcqqGKbazaeaacqqGqbaucqqGVbWBcqqG3bWDcqqGLbqzcqqGYbGCcqGH9aqpdaWcaaqaaiabigdaXaqaaiabiwda1iabicdaWiabicdaWiabgEna0kabd2gaTnaaBaaaleaacqaIXaqmaeqaaaaaaaGcdaaeWbqaaiabdsfaunaaBaaaleaacqWGRbWAaeqaaaqaaiabdUgaRjabg2da9iabigdaXaqaaiabiwda1iabicdaWiabicdaWaqdcqGHris5aOGaeiOla4caaa@66CA@

In practice, it is possible that multiple genes are ranked in the same layer selected for differential expression. In order to eliminate ties and select the same number of top ranked genes as the p-value ranking, we calculated a secondary score based on the average of ranks of - log_10_(p-value) and fold-change for each gene in the last layer. We then selected genes with the largest scores. The secondary score is a measure that have incorporated the magnitudes of the p-value and fold-change rankings.

Table 4 shows the estimated FDR's and powers from the univariate p-value ranking and two-dimensional layer rankings for the independent model (*ρ *= 0). The estimated FDR's from the p-value ranking are generally approximately close to the 5% significance level with several exceptions when *n *= 10. In particular, the FDR is 5.4% when *n *= 10 for *δ *= 1.0 and 1.2. The FDR estimates from the layer rankings have the FDR of 5.1% or 5.2% in several cases when *δ *is greater than 2 (a 4 fold-change). The estimated powers from the three layer rankings are all higher than the powers from the p-value ranking. The convex ranking appears to perform more consistent than either the point-admissible or Pareto ranking in maintaining the FDR level. The FDR estimates are all below the significance level for *δ *≤ 2. The simulation results for the correlated models (Tables 5 and 6) are similar to the results of the independent model (Table 4). The estimated FDR's from the p-value ranking are close to the significance 5% level with few exceptions when *n *= 10 and *δ*'s are small. The convex layer ranking performs well in terms of maintaining the FDR level.

**Table 4:** Simulation Results for the Independent Models

			FDR				Power			
				
*n*	*m*_1_	*δ*	P-val	P-adm	Convex	Pareto	P-val	P-adm	Convex	Pareto
10	50	1.0	5.4	3.7	3.4	3.4	2.1	2.8	2.8	2.9
		1.2	5.4	3.3	3.0	2.8	7.0	8.3	8.3	8.7
		1.4	5.1	3.0	2.9	2.7	20.8	22.5	23.0	24.0
		1.6	5.0	3.0	3.0	2.9	40.4	42.2	43.2	44.3
		1.8	4.7	2.8	2.8	2.8	62.1	64.2	65.2	65.8
		2.0	4.9	2.9	3.1	3.1	77.9	80.3	81.2	81.9
		2.5	5.1	4.6	4.6	5.1	96.4	97.9	98.0	98.2
		3.0	5.0	5.1	5.1	5.2	99.7	100.0	100.0	100.0
	100	1.0	5.0	2.9	2.8	2.5	3.3	3.9	4.0	4.2
		1.2	5.2	3.0	3.3	3.1	13.9	14.9	15.2	15.8
		1.4	4.7	2.7	2.9	2.8	34.4	35.7	36.2	36.9
		1.6	4.5	2.7	2.9	2.8	56.1	57.7	58.4	59.1
		1.8	4.5	2.8	3.0	2.8	74.5	76.3	76.9	77.5
		2.0	4.7	3.0	3.3	2.8	87.0	88.9	89.2	89.6
		2.5	4.8	4.3	4.5	4.9	98.3	99.2	99.3	99.3
		3.0	4.6	5.1	5.1	5.2	99.9	100.0	100.0	100.0
										
15	50	1.0	4.2	3.1	3.3	3.1	13.6	15.0	15.1	15.7
		1.2	4.6	3.1	3.2	3.3	37.4	38.9	39.7	40.6
		1.4	4.8	3.3	3.5	3.5	63.5	65.5	66.2	66.8
		1.6	4.9	3.6	3.8	4.0	82.8	84.6	85.2	85.7
		1.8	4.9	4.2	4.3	4.9	93.3	94.7	95.1	95.2
		2.0	4.7	4.8	4.9	5.1	97.7	98.6	98.7	98.7
		2.5	4.5	5.0	4.9	5.1	99.9	100.0	100.0	100.0
		3.0	4.7	5.1	5.0	5.2	100.0	100.0	100.0	100.0
15	100	1.0	4.7	3.1	3.2	3.1	22.3	23.2	23.6	24.2
		1.2	4.6	3.1	3.3	3.3	50.0	51.2	51.8	52.4
		1.4	4.6	3.3	3.5	3.6	74.2	75.7	76.2	76.7
		1.6	4.5	3.4	3.7	3.8	89.0	90.3	90.7	90.9
		1.8	4.5	3.9	4.1	4.3	96.0	97.1	97.2	97.3
		2.0	4.5	4.5	4.6	4.9	98.8	99.3	99.4	99.4
		2.5	4.6	5.0	5.1	5.2	100.0	100.0	100.0	100.0
		3.0	4.5	5.1	5.0	5.2	100.0	100.0	100.0	100.0

The average FDR and power estimates from 500 simulations at the FDR level of 5%. The same number of genes is selected using the Benjamini and Hochberg procedure from the p-value ranking at FDR = 5%. All values are in percentages.

**Table 5:** Simulation Results for the Correlated Models with *ρ *= 0.3

			FDR				Power			
				
*n*	*m*_1_	*δ*	P-val	P-adm	Convex	Pareto	P-val	P-adm	Convex	Pareto
10	50	1.0	4.9	3.2	3.1	3.0	2.2	3.0	2.9	3.0
		1.2	5.3	3.4	3.1	3.1	7.7	9.0	9.1	9.5
		1.4	4.8	2.8	2.8	2.7	20.3	21.9	22.4	23.1
		1.6	5.2	3.1	3.2	3.0	41.0	43.1	43.9	44.9
		1.8	4.7	2.8	3.0	2.9	61.3	63.6	64.3	65.3
		2.0	4.9	3.0	3.2	3.3	77.4	79.9	80.5	81.2
		2.5	4.9	4.5	4.6	4.9	96.4	97.9	98.0	98.1
		3.0	4.7	5.0	4.9	5.1	99.7	99.9	99.9	99.9
	100	1.0	4.6	3.0	2.8	3.0	3.8	4.5	4.5	4.7
		1.2	5.1	2.6	2.6	2.6	14.1	15.1	15.4	16.0
		1.4	4.7	2.8	3.0	2.9	33.1	34.3	34.9	35.6
		1.6	4.8	2.8	3.0	3.0	56.7	58.3	59.0	59.6
		1.8	4.5	2.8	2.9	2.9	74.5	76.3	76.9	77.4
		2.0	4.6	3.1	3.3	3.3	86.6	88.4	88.8	89.2
		2.5	4.5	4.2	4.4	4.7	98.4	99.2	99.3	99.3
		3.0	4.6	5.0	5.0	5.2	99.9	100.0	100.0	100.0
										
15	50	1.0	5.0	4.0	3.7	3.8	13.7	14.9	15.2	15.6
		1.2	4.8	3.3	3.4	3.5	36.7	38.3	38.9	39.6
		1.4	4.5	3.1	3.3	3.3	61.5	63.3	64.0	64.8
		1.6	4.8	3.6	3.7	3.9	82.3	84.0	84.8	85.1
		1.8	4.9	4.2	4.4	4.7	93.1	94.4	94.9	94.9
		2.0	4.8	5.0	5.0	5.4	97.6	98.5	98.5	98.6
		2.5	4.5	4.8	4.7	5.1	99.9	100.0	100.0	100.0
		3.0	4.7	5.1	4.9	5.2	100.0	100.0	100.0	100.0
15	100	1.0	4.6	3.3	3.2	3.2	22.5	23.4	23.9	24.5
		1.2	4.4	3.1	3.3	3.3	49.6	50.7	51.4	51.8
		1.4	4.6	3.2	3.5	3.4	74.0	75.5	75.9	76.3
		1.6	4.3	3.3	3.5	3.6	89.0	90.3	90.6	90.9
		1.8	4.5	4.1	4.3	4.6	96.1	97.0	97.1	97.2
		2.0	4.5	4.5	4.6	4.9	98.9	99.4	99.4	99.4
		2.5	4.4	5.1	5.1	5.2	100.0	100.0	100.0	100.0
		3.0	4.4	5.1	5.1	5.2	100.0	100.0	100.0	100.0

The average FDR and power estimates are same as in Table 4.

**Table 6:** Simulation Results for the Correlated Models with *ρ *= 0.6

			FDR				Power			
				
*n*	*m*_1_	*δ*	P-val	P-adm	Convex	Pareto	P-val	P-adm	Convex	Pareto
10	50	1.0	4.2	3.0	2.8	2.7	2.5	3.0	3.1	3.2
		1.2	4.1	3.2	3.1	3.1	8.3	9.4	9.4	9.8
		1.4	5.5	3.1	3.1	3.0	22.0	23.6	24.1	24.8
		1.6	5.1	3.2	3.2	3.1	39.3	41.2	41.9	42.7
		1.8	5.0	3.1	3.2	3.2	61.9	64.0	64.9	65.7
		2.0	5.0	3.3	3.5	3.7	77.6	79.7	80.3	81.0
		2.5	4.5	4.4	4.4	4.8	96.1	97.2	97.4	97.5
		3.0	4.5	5.0	5.0	5.1	99.7	99.8	99.9	99.9
	100	1.0	5.2	3.6	3.1	3.2	4.4	5.1	5.1	5.3
		1.2	4.2	2.5	2.6	2.5	14.4	15.3	15.6	16.1
		1.4	3.9	2.4	2.5	2.4	32.2	33.5	34.0	34.7
		1.6	4.7	2.9	3.0	3.0	55.9	57.5	58.1	58.7
		1.8	4.5	2.9	3.1	3.1	73.8	75.4	76.0	76.5
		2.0	4.4	2.9	3.2	3.2	86.0	87.7	88.2	88.4
		2.5	4.5	4.4	4.6	4.9	98.4	99.1	99.2	99.2
		3.0	4.5	4.8	4.8	5.1	99.9	100.0	100.0	100.0
										
15	50	1.0	5.1	3.7	3.7	3.8	15.5	16.6	16.9	17.3
		1.2	4.7	3.3	3.5	3.5	36.6	38.3	38.8	39.6
		1.4	4.6	3.3	3.4	3.6	61.7	63.6	64.3	64.8
		1.6	4.8	3.6	3.6	4.0	81.2	83.1	83.5	83.9
		1.8	4.5	4.0	4.2	4.4	92.6	93.8	94.1	94.3
		2.0	4.6	4.8	4.9	5.1	97.5	98.2	98.3	98.3
		2.5	4.8	5.1	5.0	5.3	100.0	100.0	100.0	100.0
		3.0	4.8	5.2	5.0	5.3	100.0	100.0	100.0	100.0
	100	1.0	4.3	2.9	2.9	2.9	23.2	24.1	24.5	24.9
		1.2	4.3	3.3	3.4	3.4	49.4	50.4	51.0	51.6
		1.4	4.6	3.5	3.7	3.7	73.7	75.0	75.6	76.0
		1.6	4.3	3.3	3.6	3.7	89.1	90.4	90.6	90.9
		1.8	4.5	4.1	4.2	4.4	95.8	96.7	96.8	96.9
		2.0	4.6	4.7	4.8	5.1	98.8	99.2	99.3	99.3
		2.5	4.4	5.1	5.0	5.2	100.0	100.0	100.0	100.0
		3.0	4.6	5.2	5.1	5.3	100.0	100.0	100.0	100.0

The average FDR and power estimates are same as in Table 4.

Tables 4, 5, 6 show that all three layer rankings exhibit higher power than the p-value ranking. However, the FDR estimates from the Pareto ranking often exceed the significance level when *δ *is greater than 2. As discussed, the point-admissible ranking generally produces the most layers, while the Pareto produces the fewest layers. Genes with high p-value rankings or high fold-change rankings will likely be ranked higher by the Pareto than by either the point-admissible or convex ranking. For example, a non-differentially expressed gene may have a large fold-change because of a large variance. This gene is likely be ranked higher, and be selected as differential expression by the Pareto ranking than by the point-admissible or convex ranking. Likewise, a differentially expressed gene is more likely to be selected by the Pareto ranking than by either the point-admissible or convex ranking. With regard to the point-admissible and convex rankings, their FDR estimates are all below the significance level when *δ *≤ 2. The convex ranking gives a slightly higher power than the point admissible ranking.

As seen in Tables 4, 5, 6, the FDR estimates from the p-value ranking can exceed the significance level for small effect sizes when the sample size is 10. On the other hand, the FDR estimates from the layer rankings increase as the effect size *δ *increases when the sample size is 15. In general, when the effect sizes are large (equivalently, the sample sizes are large), the power of the t-statistic approaches to 1. The BH procedure would select all truly differentially expressed genes from the p-value ranking while maintaining the FDR at the significance level. That is, the p-value ranking will outperform the layer rankings in terms of the control of the FDR, particularly, when the non-differentially expressed genes have large variances. Finally, we do not consider the null model of no difference between two groups. Since the BH procedure controls the FDR, by selecting the same number of genes the layer ranking procedures will have the FDR controlled under the null model.

### Three Applications

#### (1) Colon Data set

We further used the colon cancer data to illustrate a possible application of the layer ranking algorithms for improving predictive accuracy in classification. There were three univariate ranking criteria, three 2-dimensional layer rankings, and one 3-dimensional layer rankings. For each ranking criterion, the top 8, 16, and 32 ranked genes were used for prediction using the SVM classification (without gene selection). In order to select a pre-specified number of genes (8, 16, or 32) the gene(s) from the last layer was randomly selected when there are ties. For each gene set, we used the 10-fold cross validation to estimate predictive accuracy. Note that cross-validation performed after gene selection process is known as internal cross-validation (e.g., the SVM classifier), whereas cross-validation prior to gene selection is known the external cross-validation [8]. For a fixed number of genes, the internal cross-validation should have higher accuracy rates than the external cross-validation.

Table 7 shows the predictive accuracy rates for fifteen ordering criteria. It can be seen that (1) the more number of genes used the higher accuracy rates can be reached; (2) frequency criterion outperforms p-value and fold-change criteria in general; (3) two-dimensional rankings for p-value and fold-change in general outperforms their corresponding univariate rankings; (4) two-dimensional Pareto ranking improves prediction accuracy for frequency using 16 genes.

**Table 7:** Classification Results for Colon Data set

	Number of Genes		
	
Ranking	8	16	32
Univariate (One-Dimensional) Ranking			
			
P-value (P-val)	84.4	88.8	88.6
Fold-Change (FC)	86.9	87.3	88.5
Frequency (Freq)	89.6	89.9	91.9
			

Two-Dimensional Ranking			
			
P-val & FC (1)	88.7	88.8	90.0
P-val & FC (2)	88.7	88.7	88.8
P-val & FC (3)	86.6	88.7	88.7
			
P-val & Freq (1)	88.9	89.7	89.9
P-val & Freq (2)	89.1	86.7	90.5
P-val & Freq (3)	89.8	90.4	90.6
			
FC & Freq (1)	88.3	89.9	91.4
FC & Freq (2)	87.2	88.1	91.9
FC & Freq (3)	88.7	90.0	91.5

Three-Dimensional Ranking			
			
P-val & FC & Freq (1)	86.3	89.0	90.3
P-val & FC & Freq (2)	88.4	89.1	89.8
P-val & FC & Freq (3)	88.7	86.7	90.5

Gene ID, p-value, fold-change, and the layer assigned by the (1) point-admissible, (2) convex, and (3) Pareto algorithms for 70 genes selected based on the p-value 0.001 and 2.0-fold as the cutoff from the 2,000 genes in the colon data set. The p-values are computed based on 50,000 permutations.

#### (2) Ionizing Radiation Data set

In this example, we used the layer ranking algorithms to identify genes that show most differentially expressed in two experimental factors. The experiment was conducted to study the effects of ionizing radiation-exposed human lymphoblastoid TK6 cells on gene expression [13]. In this experiment, TK6 cells were exposed to 5, 10, and 20 Gy ionizing radiation and cultured for 4 and 24 hours after exposure. RNA was hybridized to the Phase-1 Human-350 microarray (Phase 1 Molecular Toxicology, Santa Fe, NM) spotted with 350 human cDNA probes. This two-color array was designed for detection of differential expression profiles relative to toxicological pathways. The background-subtracted intensities were normalized according to Lowess methodology in the log_2 _scale, and a dye-bias correction was applied to the resulting data, as described in [14].

For illustrative purpose, we only used the 5 and 10 Gy ionizing radiation data. Statistical analysis consisted of a comparison between the 5 and 10 Gy dose groups and a comparison between the 4 and 24 hours after exposure. In each comparison, the differentially expressed genes were ranked according to the p-values using the permutation t-test. The layer ranking algorithms were applied to both p-value rankings. Table 8 shows the top 10 layers, according to the point-admissible algorithm, for the three layer rankings and the two p-value rankings, where *P*_1 _and *P*_2 _represent the ranking for the dose and time effects, respectively. It can be seen that dose and time effects show two distinct rankings. Each p-value ranking identifies a set of genes that show differences in expression on a single biological factor. The layer rankings provide a list of 'significant' genes that account for both dose and time effects simultaneously. Many of the top hits have been reported to be important in lymphoblastoid cell lines exposed to ionizing radiation provides confidence that the analysis produces meaningful results.

**Table 8:** Rankings on two p-values for Ionizing Radiation Data set

Gene name	P-A	Convex	Pareto	*P*_1_	*P*_2_
Carcinoembryonic antigen (CD66e)	1	1	1	2	22
Gadd45	1	1	1	1	262
Glucose-6-phosphate dehydrogenase	1	1	1	12	2
p55CDC	1	1	1	3	10
Uncoupling protein-2	1	1	1	55	1
Heat shock protein-90	2	3	3	30	5
RAD 51 homologue	2	2	2	14	8
RANTES	2	2	2	4	14
Phenol sulfotransferase	3	2	2	96	3
Glutathione peroxidase	4	4	4	45	9
Pim1 proto-oncogene	4	3	3	127	4
SSAT	4	3	3	6	23
Transthyretin	4	3	3	5	60
Transferrin	5	6	6	29	21
Biliary glycoprotein	6	6	6	10	151
Casein kinase 1 delta	6	5	5	8	177
Interferon stimulatory gene factor-3	6	4	4	178	6
Plasminogen activator inhibitor-2	6	4	4	13	31
c-jun	7	6	6	9	255
Carnitine palmitoyl-CoA transferase	7	5	5	95	12
Heme oxygenase-1	7	5	5	318	7
Multidrug resistant protein-1	7	4	4	7	274
Neurofibromin (NF1 tumor suppressor)	7	5	5	19	29
Connexin-40	8	6	6	16	58
STAT-3	8	8	8	80	17
Activating transcription factor-3	9	8	8	20	47
Cell division cycle protein-25	9	6	6	174	11
Hypoxanthine-guanine phosphoribosyltransferase	9	7	7	23	36
S-adenosylmethionine decarboxylase	9	7	7	32	25
Alpha-1 acid glycoprotein	10	9	9	18	90
Bcl-xL	10	7	7	148	13
FosB	10	7	7	103	15
Tryptophanyl-tRNA synthetase	10	8	8	27	38

Gene name, point-admissible (P-A), Convex, and Pareto layer rankings, and the p-value (*P*_1 _and *P*_2_) rankings for the top 33 genes selected based on the point-admissible ranking from the 350 genes in the ionizing radiation data set.

#### (3) Dilution Data set

In this example, we applied the three-dimensional layer ranking criteria to a subset of the dilution data set of Gene Logic and the data are available at [15]. This study used two sources of cRNA, human liver tissue and central nervous system (CNS) cell lines. Samples were hybridized to HG-U95Av2 GeneChips arrays from Affymetrix at various dilution and mixture levels. We considered the data from the three concentrations 7.5, 10.0, and 20.0 *μg*. Five replicate arrays are available for each concentration with a total of 30 arrays. The data were extracted, normalized and summarized using the "Affy" package from Bioconductor. For preprocessing methods, we used MAS 5.0 for background correction and PM correction, the quantile normalization method for normalization of the probe level, and the RMA method for summarization of probe intensities, which was suggested by [16].

Since the two samples are biologically distinct, it is expected that many genes will show differential expressions between the two samples. The relative abundance of each gene is proportional to its dilution concentration. However, the expression ratios between the pure samples (fold changes) are relative and should not vary with the amount of cRNA. If a gene expresses differently between the liver and CNS samples at 20.0 *μg *concentration, then we expect the same gene would show a difference in expression at other concentrations. In this application, the layer ranking algorithm is used to provide a ranking system from a long list of differentially expressed genes generated from three dilution concentrations.

**Figure 2:**
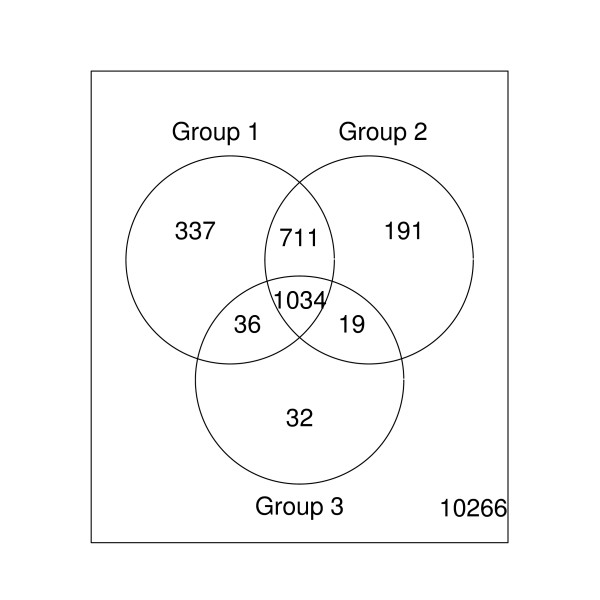
**Results of Dilution Data Set**. Venn diagram of the dilution data set demonstrating the agreement in the number of differentially expressed genes selected from the three concentrations using SAM procedure at the 0.01 FDR level.

**Figure 3:**
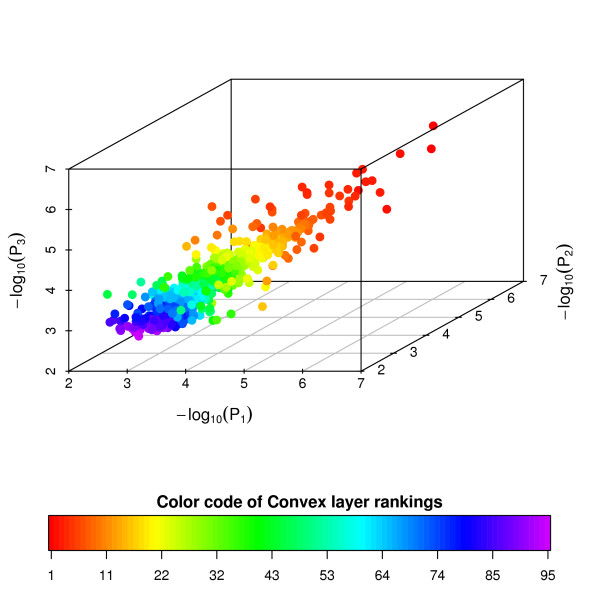
**3D convex layer ranking for Dilution Data Set**. Scatter plot of three p-value lists for the 1034 differentially expressed genes selected from Figure 2. Colors represent the layers of genes identified by the three-dimensional convex layer ranking algorithm.

The liver and CNS samples were compared for each of the three concentrations using the SAM procedure [17]. The level of significance was set at the false discovery rate of 0.01. Let *G*_1_, *G*_2_, and *G*_3 _be the sets of the differentially expressed genes identified from the concentrations 20.0, 10.0, and 7.5 *μg*, respectively. Figure 2 summaries the number of differentially expressed genes selected from the three comparisons. The overlapping regions denote the number of genes that were differentially expressed in two or all three concentration concentrations. There were 1034 genes which show consistently differential expressions in the two RNA samples in all three concentrations. We then applied the three-dimensional convex layer ranking algorithm to three p-value lists obtained from the SAM procedure. Ninety-five layers were obtained. The layer rankings of 1034 differentially expressed genes are shown in Figure 3. The other two layer ranking algorithms yield similar results (data not shown).

Table 9 provides the Kenall's *τ *correlation matrix of the p-value rankings of 1034 genes for the three concentrations with the three proposed three-dimensional layer rankings. Ideally, the p-value rankings of the 1034 genes identified as changes in expression between the liver and CNS samples should have high agreement across the three RNA concentrations. It is apparent that there are many discordances among the 20 *μg *10 *μg*, and 7.5 *μg *p-value rankings (Figure 3 and Table 9). In contrast, the three layer ranking algorithms improve the comparability (Table 9); they provide compromising gene rankings by combining information across the three RNA concentrations. The layer ranking algorithms can help an investigator to pick a few of top-ranked genes with confidence across multiple listings for further investigation.

**Table 9:** Kendall's *τ *correlation among six rankings

	20.0 *μg*	10.0 *μg*	7.5 *μg*	P-A	Convex	Pareto
20.0 *μg*	1.000					
10.0 *μg*	0.695	1.000				
7.5 *μg*	0.660	0.669	1.000			
P-A	0.776	0.794	0.812	1.000		
Convex	0.765	0.789	0.824	0.978	1.000	
Pareto	0.769	0.794	0.825	0.978	0.985	1.000

Kendall's *τ *correlation matrix among six rankings, three p-value rankings from three concentrations and three three-dimensional layer rankings, for the 1034 genes showing consistenly differential expression in all three concentrations in the Dilution data set.

## Discussion and Conclusion

Recently, the MicroArray Quality Control consortium suggested: "Fold-change ranking plus a non-stringent P-value cutoff can be used as a baseline practice for generating more reproducible signature gene lists" [18]. Many researchers have questioned this approach [19]. The p-value ranking ensures the control of significance level, while the fold-change ranking may provide a better ranking when sample size (or effect size) is small. The layer-ranking algorithms provide a gene list that reconciles the p-value and fold-change rankings implied by the volcano plot. The simulation shows that for experiments with small or moderate sample sizes (less than 20 per group) and detecting a 4-fold change or less, the layer ranking selects differentially expressed genes with generally lower FDR and higher power than the p-value ranking. For large sample sizes or effect sizes, the p-value ranking will outperform the layer rankings.

We illustrate three additional applications of the layer ranking algorithms. In the colon data example, we illustrate an application of using layer rankings for improving predictive accuracy. Because of a large number of genes involved, the gene selection becomes one of the most important steps in the development of a prediction model. An analysis by Michiels et al. [20] showed that the list of genes identified as predictors of cancer prognosis was highly unstable. The selected gene set strongly depended on the selected patients in the training set. In this example, we consider the three gene selection criteria: p-value, fold-change, and frequency of selections. Table 8 indicates that the improvement of predictive accuracy of the layer rankings over the p-value rankings appears marginal. The simulation indicates that when the sample size is large, the layer rankings can exceed the significant level. This may not be a problem for prediction purposes, since the omission of informative genes generally has a much more serious consequence on predictive accuracy than the inclusion of non-informative genes. We are currently investigating different univariate selection criteria in conjunction with layer ranking algorithms to improve predictive accuracy. In general, the p-value can be calculated in many different ways such as the parametric t-test, permutation t-test, or SAM method. Frequency of selections can be calculated by other classification algorithms such as CTree [2] or Random Forest [21]. In addition, instead of selecting 8 optimal genes in each cross validation, we may select 64 genes or more so that each gene has higher probability been selected. Those genes that have never been selected are unlikely to be differentially expressed. The 2-dimensional p-value and frequency layer rankings may be useful to filter out a small number of non-differentially expressed genes. The genes in the bottom layers may be the candidates for filtering out.

In the ionizing radiation example, we apply the layer ranking algorithms to a two-factor experiment. The algorithms can be used in the one-factor experiment with more than two conditions. Consider an experiment to study effect of p53 genotype on gene expression profiles. The experiment consists of three mouse genotypes: wild-type (+/+), knock-out (-/-), and heterozygous (+/-). Statistical analysis typically consists of a comparison among the three genotypes. A gene list ranked according the p-values from the F-statistic can be obtained using either permutation or parametric approach. An important follow-up analysis is the comparisons between the knout-out and wild-type mouse and between the heterozygous and wild-type mouse. The Dunnett's test is frequently used to generate the differentially expressed gene lists for the two comparisons. However, the investigator is often interested in the genes that show differences in both comparisons. (Note that the significant genes identified in the F-test may be insignificant in both Dunnett's tests.) One approach is to select the genes that are significant in both gene lists at a given p-value cutoff. However, when the number of common genes is large, the investigator must select a subset of genes from the two criteria (the dilution example). The layer ranking algorithm can be used to provide a list of the most "important" genes that account for both objectives simultaneously for follow-up investigation. In the dilution example, we illustrate the strength of the three-dimensional layer ranking algorithms for combining discordant results derived from three concentration groups. The set of probes that are consistently identified at different RNA concentrations is ranked according to compatibility between differential expression profiles in three concentration groups.

In summary, a microarray experiment can generate different gene lists by different filter, normalization, or analysis methods for different study objectives. The layer ranking algorithm can be useful to help investigators to select the most promising genes from multiple gene lists.

## Methods

Let *S *= {*p*_*i *_= (*x*_*i*_, *y*_*i*_) | *i *= 1,..., *m*} denote the set of points under consideration, where *x*_*i *_> 0. For example, *x *is the fold change in absolute value and *y *is (-log_10 _*p*). Barndorff-Nielsen and Sobel [22] proposed a layer ranking criterion for ordering multivariate data. The layer ranking divides *S *into disjoint sets (layers) of different ranks, the points in the same layer have the same rank. In this paper, we present three layer ranking criteria based on the principle of the first quadrant-admissible [22]. A point, *p*_*i *_= (*x*_*i*_, *y*_*i*_), is called first quadrant-admissible in *S *if there does not exist any point *p *= (*x*, *y*) such that *x *> *x*_*i *_and *y *> *y*_*i*_. Conversely, a point, *p*_*i *_= (*x*_*i*_, *y*_*i*_), is dominated by another point *p*_*j *_in *S *if (*x*_*i *_<*x*_*j *_and *y*_*i *_≤ *y*_*j*_) or (*x*_*i *_≤ *x*_*j *_and *y*_*i *_<*y*_*j*_). Three layer ranking algorithms are described below.

### Point-admissible layer

A point (*x*_*i*_, *y*_*i*_) is called *r*-th layer (first quadrant) admissible (*r *= 1,2, ...) [22] if there are exactly (*r *- 1) points (*x*, *y*) such that *x *> *x*_*i *_and *y *> *y*_*i*_. Let *S*_*r *_denote the set of *r*-th layer admissible (*r *= 1, 2,...). Each observation is either *r*-th layer admissible (*r *= 1, 2, ...) or inadmissible; that is, *S *= *S*_1 _∪ *S*_2 _∪ ... . For each point *p*_*i *_= (*x*_*i*_, *y*_*i*_) in *S*, let *r *denote the number of points *p *= (*x*, *y*)'s for which *x *> *x*_*i *_and *y *> *y*_*i*_. The point *p*_*i *_is assigned to the (*r *+ l)-th layer (*r *= 0,1,...).

### Line-admissible (convex) layer

A line segment is called (first quadrant) admissible in *S *if every point on the line segment is first quadrant-admissible in *S*. The 1-st line-admissible layer is obtained by finding the admissible points that are connected by line(s) with non-positive or infinite slopes (the minimum convex set). The *r*-th layer is obtained similarly by stripping off the points on the (*r *- 1)-th layers (*r *= 2, 3...).

### Pareto layer

The Pareto layer (front) was introduced by [23] for gene ranking. The 1-st Pareto layer consists of all points not dominated by other points. The *r*-th layer is obtained similarly by striping off the points on the (*r *- l)-th layers (*r *= 2, 3...).

**Figure 4:**
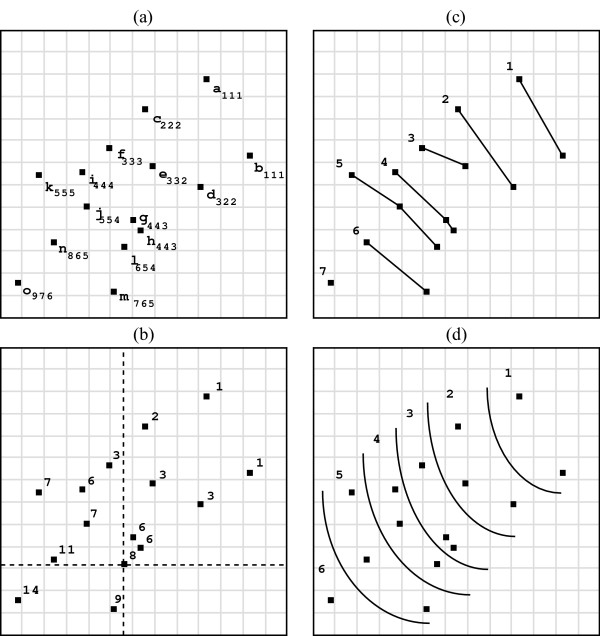
**Three Ranking Methods**. Illustration of three layer ranking algorithms for 15 points. (a) Fifteen data points (a-o) on a two-dimensional space. The subscripts represent the layers for which the point is assigned by the three algorithms. For example, the subscript 865 indicates that point n is assigned to 8th, 6th and 5th layers for (b) Point-admissible algorithm, (c) Convex algorithm, and (d) Pareto algorithm respectively.

The three layer ranking algorithms are illustrated in Figure 4. Figure 4(a) consists of 15 points (a-o). In the point admissible layer ranking, for a given point, say, *l*, the number of points with both coordinates greater than *l *(the number of points in the upper right) is 7. The point *l *is assigned to the 8-th layer (Figure 4(b)). Note that the *point admissible layer *may have empty layers, for example, 4-th and 5-th layers. Figures 4(c) and 4(d) show the *line-admissible layers *and Pareto layers, respectively. In Figure 4(c), all line segments have negative slopes. The point *e *is dominated by the line segment connected points *c *and *d*. Therefore, *e *is assigned to the next (3-rd) layer. In Figure 4(d), *e *has a larger x-coordinate than *c*, and a larger y-coordinate than *d*. Therefore, *e *is not dominated by *c *or *d*; and *c*, *d*, and *e *are all assigned to the 2-nd layer. The subscripts in Figure 4(a) represent layers for which the point is assigned by the three algorithms. For example, the point n has the subscript 865 indicating that n is assigned to the 8th, 6th and 5th layers by the point-admissible, convex, and Pareto algorithms, respectively. Note that the empty layers for the r-th layer algorithm are removed and the non-empty layers are renumbered. The numbers of layers for the three ranking algorithms are 9, 7, and 6 (point o). For continuous measurements such as fold-change or p-value, the *point-admissible *ranking generally produces the most layers, while the *Pareto *ranking produces the fewest layers.

Extending the previous point-admissible layer and Pareto layer to three or more dimensional situations is straightforward. For three-dimensional examples, a point *p*_*i *_= (*x*_*i*_, *y*_*i*_, *z*_*i*_) is assigned to the (r + 1)-th layer if there are r points *p *= (*x*, *y*, *z*)'s such that *x *> *x*_*i*_, *y *> *y*_*i*_, and *z *> *z*_*i*_. Similarly, a point *p*_*i *_= (*x*_*i*_, *y*_*i*_, *z*_*i*_) belongs to the 1-st Pareto layer if no other points dominate it, i.e.,

{(*x*, *y*, *z*)|*x *≥ *x*_*i*_, *y *≥ *y*_*i*_, *z *≥ *z*_*i*_}\{(*x*, *y*, *z*)|*x *= *x*_*i*_, *y *= *y*_*i*_, *z *= *z*_*i*_}

is the null set. Then points are assigned to the r-th Pareto layer (r = 2,3,...) recursively in the same way by striping off the points on the 1-st,...,(r-1)-th layers.

To find a higher-dimensional convex layer, an algorithm for determining the convex polytope (convex hull in an arbitrary dimension) is needed and interested readers are referred to Chapter 11 of [24]. One popular implementation can also be found at [25]. The 1-st convex layer is obtained as the intersection of the points lying on the convex polytope and on the 1-st Pareto layer. The next step is to recursively strip off the points on the 1-st,..,(r-1)-th layers, and points are assigned to the r-th convex layer (r = 2,3,...) if they lie on the resultant convex polytope and on the resultant 1-st Pareto layer.

## Availability and requirements

A multiple ordering procedure for gene selection written in R with various options is freely available.

Project name: multiple ordering gene selection

Project home page: 

Operating systems: any OS that supports the R environment

Programming languages: R

License: free

## Authors' contributions

JJC and CHC conceived the study, developed the methodology, and wrote the manuscript. CHC and ST developed and implemented the main algorithm. CAT implemented the SVM algorithm and conducted the simulation experiment. CAT and ST performed the analysis. All authors read and approved the final manuscript.
